# The Mangled Extremity Severity Score (MESS) does not predict amputation in popliteal artery injury

**DOI:** 10.1007/s00068-022-02179-4

**Published:** 2022-11-30

**Authors:** Alexandra Gratl, Michaela Kluckner, Leonhard Gruber, Josef Klocker, Sabine Wipper, Florian Karl Enzmann

**Affiliations:** 1 Department of Vascular Surgery, Medical University Innsbruck, University Hospital of Innsbruck, Anichstraße 35, 6020 Innsbruck, Austria; 2 Department of Radiology, Medical University Innsbruck, University Hospital of Innsbruck, Anichstraße 35, 6020 Innsbruck, Austria

**Keywords:** Vascular trauma, Popliteal artery, Mangled Extremity Severity Score, Severity of trauma, Fasciotomy, Amputation

## Abstract

**Purpose:**

Vascular injuries in lower extremity trauma, especially with involvement of the popliteal artery, are associated with considerably high rates of limb loss, especially with blunt trauma mechanisms. The aim of this study was to evaluate the risk of amputation in patients with traumatic popliteal artery lesions with special focus on the validity of the Mangled Extremity Severity Score (MESS).

**Methods:**

In this retrospective study, all patients treated for isolated lesions of the popliteal artery following trauma between January 1990 and December 2020 at a high-volume level I trauma center were included. Primary outcome was limb salvage dependent on MESS and the influence of defined parameters on limb salvage was defined as secondary outcome. The extent of trauma was assessed by the MESS.

**Results:**

A total of 50 patients (age 39.2 ± 18.6 years, 76% male) with most blunt injuries (*n = *47, 94%) were included. None of the patients died within 30 days and revascularization was attempted in all patients with no primary amputation and the overall limb salvage rate was 88% (44 patients). A MESS ≥ 7 was observed in 28 patients (56%) with significantly higher rates of performed fasciotomies (92.9% vs. 59.1%; *p < *0.01) in those patients. MESS did not predict delayed amputation within our patient cohort (MESS 8.4 ± 4.1 in the amputation group vs. 8.1 ± 3.8 in the limb salvage group; *p = *0.765).

**Conclusion:**

Revascularization of limbs with isolated popliteal artery injuries should always be attempted. MESS did not predict delayed amputation in our cohort with fasciotomy being an important measure to increase limb salvage rates.

**Supplementary Information:**

The online version contains supplementary material available at 10.1007/s00068-022-02179-4.

## Introduction

Arterial involvement in blunt or penetrating trauma of the lower extremities is associated with considerably high rates of limb loss. With a reported frequency of 3% in all trauma cases, extremities are most commonly affected by vascular injuries [1]. Traumatic arterial involvement, concomitant destruction of adjacent bones, nerves as well as soft tissue result in complex injuries needing interdisciplinary care to optimize the patients’ outcome. The popliteal artery is known to be the second most frequently affected artery in patients with lower extremity arterial injury [2, 3] being categorized as a grade IV injury referring to the American Association for the Surgery of Trauma (AAST) Organ Injury Scale [4]. Traumatic injury of the popliteal artery is also associated with the highest risk of amputation in peripheral arterial injuries [5]. Especially in penetrating trauma mechanisms, hemorrhagic shock resulting from vascular injury is potentially life threatening. In civilian trauma, blunt trauma mechanism is the most common type of injury typically being associated with limb ischemia following dissection or complete transection of the artery resulting in considerably high rates of limb loss. Amputation rates are reported to be higher in patients with a blunt trauma mechanism compared to those with penetrating injuries being explainable by severe concomitant injuries of bones as well as soft tissue [6]. Recently published clinical guidelines on the diagnosis and management of peripheral vascular injuries underline the importance of an immediate diagnosis of vascular involvement in trauma patients [7].

In the early 1990s, several scoring systems have been implemented with the goal to predict primary amputation in mangled extremities. The scoring systems include the Mangled Extremity Severity Score (MESS) [8], the Predictive Salvage Index (PSI) [9], the Limb Salvage Index (LSI) [10], and the Nerve Injury, Ischemia, Soft-Tissue Injury, Skeletal Injury, Shock and Age of Patient (NISSA) Score which is a modified version of the MESS [11]. All of these scores have been established using retrospective analyses of trauma patients with MESS being the only scoring system also utilizing prospective data. Referring to a systematic review published by Schirò et al. in 2015, the MESS is the most commonly used scoring system for the purpose of grading severity of trauma [12]. The MESS includes information about skeletal and soft tissue injury, limb ischemia, shock and age of the patient. Within the initial publication of the score, a MESS > 7 was defined as a predictor for the need of primary amputation [8]. Data from civilian trauma series, reveal a low predictive value for limb salvage as well as primary amputation using the MESS [13, 14]. Others report the potential of the scoring system to identify patients at risk for primary amputation [15, 16]. Following this controversy, there is no clear consensus about the usage of the established scoring systems in clinical routine. There still appears to be a need of a scoring system predicting limb salvage in civilian lower extremity trauma. It is known that amputation after failed limb salvage attempt is associated with an increased risk of systemic complications leading to potential lift-threatening conditions [10, 12].

Data about blunt vascular trauma following sport, vehicle or work accidents are sparse, even though these trauma mechanisms may be predominant in most civilian trauma centers and may result in a patient cohort mainly comprising blunt trauma pattern injuries. The aim of this study was to evaluate patients with traumatic injuries of the popliteal artery, to identify factors associated with increased in-hospital amputation rates. Following a strict “limb salvage by any means” strategy within our institution, we further wanted to evaluate the MESS to predict secondary amputation rates within the analyzed patient cohort.

## Material and methods

### Data collection

In this retrospective study, all patients with surgical treatment for traumatic lesions of the popliteal artery at the Department of Vascular Surgery, University Hospital of Innsbruck, Austria—a high-volume level I trauma center—operated between January 1990 and December 2020 were included. In case of traumatic lesion of the popliteal artery, interdisciplinary decision making about treatment strategies is performed involving trauma, vascular as well as plastic surgery, if needed. Within our institution, we follow a strict “limb salvage by any means” policy. Ethical approval for this study was obtained at the local ethics committee of the Medical University of Innsbruck (EK1079/2020).

Demographic data, details on trauma mechanism (blunt or penetrating trauma), type of vascular trauma (dissection/thrombosis or transection of the popliteal artery), anatomic level of popliteal artery injury (above or below the knee), and type of arterial reconstruction were collected. Stage of acute ischemia was documented using the Rutherford classification system [17] and severity of trauma was graded using the Mangled Extremity Severity Score defining a score < 7 as mild and ≥ 7 as severe trauma [8]. Concomitant skeletal, venous, and nerval injuries as well as details on fasciotomy, if performed (primary—within the revascularization procedure or secondary—in case of postoperative development of compartment syndrome; open—incision with temporary wound closure using synthetic material or subcutaneous—primary closure of skin incision after performed fasciotomy) were assessed. Length of hospital stay and in-hospital amputation rates were assessed as well.

### Outcome measures

As primary outcome, in-hospital limb salvage (LS) dependent on MESS was defined. Secondary outcome was influence of evaluated parameters on in-hospital LS and amputation (AMP) as well as the predictive value of MESS on in-hospital LS and AMP rates.

### Statistics

All data were stored in Microsoft Excel 16.16.27 (Microsoft; Redmond, USA). Statistical software used was GraphPad Prism 8.4.3 (GraphPad Software LLC; La Jolla, USA) and WEKA 3.8.4 (University of Waikato; Waikato, NZ) [11].

Descriptive analysis for general demographic data including patient age, sex, Rutherford classification, MESS, and amputation rates was performed.

Patients were then categorized into two groups with an outcome of limb salvage (LS) or major amputation (AMP). A Mann–Whitney test was used for continuous data of both groups due to a non-Gaussian distribution; Fisher’s exact test was used for categorical variables. Invalid or missing data were not utilized for further analysis, and no imputation technique was employed.

The influence of the predictors assessed (Supplemental Table 1) on the occurrence of amputation was studied; a naïve Bayes Network with a 30-fold cross-validation was employed, and the model results included correct classification rate (CCR), mean absolute error (MAE), relative absolute error (RAE), and weighted average for the true positive rate (TP_avg_), false positive rate (FP_avg_), precision (P_avg_). To generate a predictor ranking, a gain ratio feature evaluation was used in combination with a ranker algorithm, and the odds ratios (OR) were calculated from contingency tables. In case of continuous or ordinal variables, the ideal cutoff was determined using receiver-operating characteristics (ROC) curvess and Youden’s J. Predictor properties reported include gain ratio, fasciotomy rate, and OR including 95% confidence intervals (CI). Statistical significance was considered for *p* values < 0.05. To evaluate the potential influence of trauma severity on amputation rates and other covariates, referring to literature, participants were grouped into those with a MESS < 7 and those with a MESS ≥ 7 [8]. The analysis was carried out as described above.

## Results:

### Population characteristics

A total of 50 patients were included in this retrospective study. The mean age was 39.2 ± 18.6 years with mostly male patients (*n = *38, 76%).

According to the Rutherford classification of acute limb ischemia, 16% of patients were classified as grade I, 28% as grade IIa, 24% as grade IIb, and 32% as grade III, while the average MESS was 8.1 ± 3.8. No primary amputation was performed in our cohort. Major amputation was performed in eight cases (16%) with a significantly longer hospital stay compared to the limb salvage patients (27.3 ± 28.4 vs. 62.3 ± 31.5 days, *p = *0.005). Popliteal artery injury was diagnosed using computed tomography in the majority of cases (*n = *33; 66%) followed by clinical diagnosis in 11 patients (22%) and angiography in 6 patients (12%).

### Injury patterns, revascularization techniques, and fasciotomies

The majority of patients presented with above-the-knee popliteal artery injury (*n = *36, 72%). The most frequent trauma mechanisms were sport accidents (*n = *19, 38%) followed by motor vehicle accidents (*n = *17, 34%) and work-related injuries (*n = *10, 20%). Within the remaining 4 patients, the trauma mechanism was unclear. The most common trauma type was blunt trauma (47 out of 50 patients, 94%) with dissection and thrombosis as the main vascular injury (*n = *31, 62%) compared to a complete transection of the popliteal artery (*n = *19, 38%). Venous interposition graft for arterial reconstruction was the predominant revascularization technique (47 out of 50 patients; 94%), followed by direct repair of injured artery in 2 patients and 1 case of patch angioplasty to reconstruct the injured arterial segment. Concomitant venous injury was present in 11 patients (22%) with venous reconstruction in 8 patients (72.7%) and 3 ligations of affected venous segments in the remaining 3 patients (27.3%). In 39 patients (78%), any type of fasciotomy was performed. In most of the cases, fasciotomy was performed open (*n = *27; 69.2%) and primary fasciotomy within the revascularization procedure was performed in 33 patients (84.6%). Type of fasciotomy (open/subcutaneous; primary/secondary) did not influence limb outcome.

### Limb salvage (LS) rates

LS was attempted in all patients by above-described revascularization procedures and achieved in 42 out of 50 patients (84%). In-hospital AMP was needed in 8 out of 50 patients leading to an in-hospital amputation rate of 16%. Secondary amputation was associated with a significantly longer hospital stay compared to the LS group (27.3 ± 28.4 vs. 62.3 ± 31.5 days, *p = *0.005).

### Amputation predictors

There were no significant differences regarding age between LS (38.8 ± 19.4 years) and AMP (40.9 ± 16.2 years) (*p = *0.782) or sex (*p = *0.661) even though amputation rates were somewhat higher for men (18.4%, 7 out of 38 cases) compared to women (8.3%, 1 out of 12 cases), without reaching statistical significance.

A modeling approach using a Bayes network analysis yielded a CCR of 92.0% (46 of 50 cases), MAE of 0.095, RAE of 33.6%, TP_avg_ of 92.0%, false positive rate FP_avg_ of 32.9%, P_avg_ of 92.0%, and a ROC-AUC of 0.783.

The strongest single predictor for amputation was maximum myoglobin levels (gain ratio 0.173 ± 0.011). While minimum eGFR levels (0.120 ± 0.009) and necessary hemofiltration (0.104 ± 0.021) were strong predictors for limb loss, baseline eGFR did not allow any prediction (0.0 ± 0.0). Work- and traffic-associated trauma mechanism (0.089 ± 0.004), venous trauma (0.072 ± 0.016), and need for vascular re-intervention (0.063 ± 0.018) also predisposed to amputation.

Details about patients’ characteristics and predictors for amputation are given in Tables 1 and 2.

**Table 1:** Patient characteristics

	Overall	Limb salvage	Amputation	*p* value
Patients [*n*, %]	50	42 (84.0)	8 (16.0)	–
Age [years]	39.2 ± 18.6	38.8 ± 19.4	40.9 ± 16.2	0.782^a^
Male sex [*n*, %]	38 (76.0)	31 (73.8)	7 (87.5)	0.661^b^
MESS (mean ± SD)	8.1 ± 3.8	8.1 ± 3.8	8.4 ± 4.1	0.830^a^
Type of trauma [*n*, %]				0.044^b^
Blunt trauma	47 (94.0)	39 (83.0)	8 (17.0)	
Penetrating trauma	3 (3.0)	3 (100.0)	0 (0.0)	
Mechanism of trauma [*n*, %]				0.044^b^
Sport accident	19 (38.0)	19 (100.0)	0 (0.0)	–
Motor vehicle accident	17 (34.0)	12 (70.6)	5 (29.4)	–
Work-related accident	10 (20.0)	7 (70.0)	3 (30.0)	–
Mechanism unclear	4 (8.0)	4(100.0)	0 (0.0)	–
Rutherford categories of ischemia [*n*, %]				0.219^b^
Grade I	8 (16.0)	7 (16.7)	1 (12.5)	–
Grade IIa	14 (28.0)	14 (33.3)	0 (0.0)	–
Grade IIb	12 (24.0)	9 (21.4)	3 (37.5)	–
Grade III	16 (32.0)	12 (28.6)	4 (50.0)	–
Ischemia time > 6 h [*n*, %]	19 (30.6)	15 (36.6)	4 (50.5)	0.694^b^
Delayed revascularization (> 12 h after trauma) [n,%]	8 (16.3)	8 (19.0)	0 (0.0)	0.854^b^
Vascular injury type [*n*, %]				0.686^b^
Dissection/thrombosis	31 (62.0)	26 (62.9)	5 (62.5)	–
Transection	19 (38.0)	16 (38.1)	3 (37.5)	–
Popliteal segment [*n*, %]				0.805^b^
Above the knee	36 (72.0%)	33 (92.0%)	3 (8.0%)	
Below the knee	14 (28.0%)	11 (79.0%)	3 (21.0%)	
Concomitant venous injury [*n*, %]	11 (22.0)	7 (16.7)	4 (50.0)	0.059^b^
Concomitant neural injury [*n*, %]	17 (34.0)	14 (33.3)	3 (37.5)	> 0.999^b^
Concomitant bone injury [*n*, %]	45 (90.0)	38 (90.5)	7 (87.5)	> 0.999^b^
Vascular re-intervention [*n*, %]	5 (6.3)	3 (7.3)	2 (28.6)	0.148^b^
Fasciotomy [*n*, %]	39 (78.0)	32 (76.2)	7 (87.5)	0.666^b^
Hospital stay (days, mean ± SD)	32.3 ± 30.8	27.3 ± 28.4	62.3 ± 31.5	0.005^a^

^a^Mann–Whitney test

^b^Fisher’s exact test

**Table 2:** Ranked predictors for amputation in popliteal artery injury by a gain ratio analysis

Predictor (cutoff)	Rank	Gain ratio	Amputation rate [%]	OR [95% CI]	*p* value
Max. myoglobin level [nMol/L] (> 5024 nMol/L)	1.1 ± 0.56	0.173 ± 0.011	18.2 vs. 0.0%	Infinity [0.33 to infinity]	0.5717
Min. eGFR [mL/min/1.73m^2^] (< 55 mL/min/1.73m^2^)	4.1 ± 0.62	0.120 ± 0.009	100.0 vs. 10.6%	Infinity [5.54 to infinity]	0.0029
Hemofiltration necessary (yes)	6.3 ± 6.66	0.104 ± 0.021	100.0 vs. 13.0%	Infinity [5.54 to infinity]	0.0029
Trauma mechanism (traffic, work)	6.4 ± 0.71	0.089 ± 0.004	29.6 vs. 0.0%	Infinity [2.14 to infinity]	0.0050
Venous trauma (yes)	7.9 ± 2.43	0.072 ± 0.016	36.4 vs. 10.3%	5.00 [1.17–20.14]	0.0591
Vascular revision (yes)	9.3 ± 4.58	0.063 ± 0.018	40.0 vs. 11.6%	5.01 [0.72–28.70]	0.1483
Interposition graft (no)	11.7 ± 3.45	0.052 ± 0.011	29.4 vs. 9.1%	4.17 [0.84–17.14]	0.1022
Rutherford classification (> IIa)	11.7 ± 1.51	0.048 ± 0.004	25.0 vs. 4.5%	7.00 [1.0–82.05]	0.0638
Limb ischemia (yes)	12 ± 1.91	0.048 ± 0.004	17.0 vs. 0.0%	Infinity [0.16 to infinity]	> 0.9999

### Prediction of amputation using the Mangled Extremity Severity Score (MESS)

**Fig. 1:**
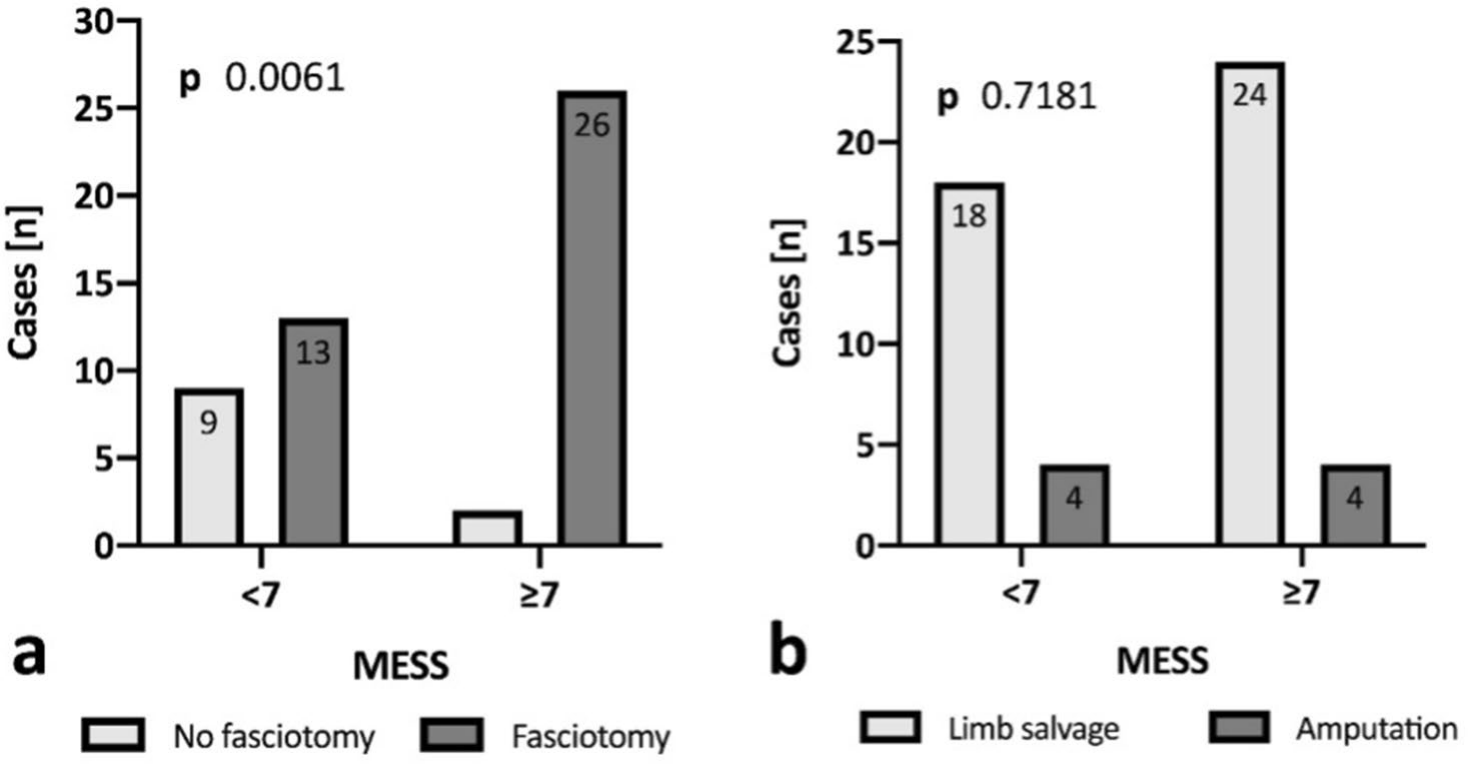
Comparison of fasciotomy (**a**) and amputation frequencies (**b**) grouped by a Mangled Extremity Severity Score (MESS) of < 7 or ≥ 7

MESS did not predict in-hospital amputation as average values for AMP (8.4 ± 4.1) and LS (8.1 ± 3.8) were comparable (*p = *0.765). When comparing patients with a MESS < 7 with those with ≥ 7, there were no significant differences regarding age, sex, arterial vascular injury, and need for vascular re-intervention. Overall fasciotomy rates were significantly higher in patients with a MESS ≥ 7 (59.1 vs. 92.9%, *p = *0.006) while amputation rates were comparable (18.2 vs. 14.3%, *p = *0.718) (Fig. 1). Further group details are provided in Table 3.

**Table 3:** Comparison between patients grouped by MESS (Mangled Extremity Severity Score)

	Overall	MESS < 7	MESS ≥ 7	*p* value
Patients [*n*, %]	50	22 (44.0)	28 (56.0)	–
Age [years]	39.2 ± 18.6	42.7 ± 19.9	36.4 ± 17.7	0.238 ^a^
Male sex [*n*, %]	38 (76.0)	18 (81.8)	20 (71.4)	0.512 ^b^
Popliteal transection [*n*, %]	19 (38.0)	6 (27.3)	13 (46.4)	0.242 ^b^
Fasciotomy [*n*, %]	39 (78.0)	13 (59.1)	26 (92.9)	0.006 ^b^
Vascular re-intervention [*n*, %]	5 (10.0)	3 (13.6)	2 (7.7)	0.649 ^b^
Amputation [*n*, %]	8 (16.0)	4 (18.2)	4 (14.3)	0.718 ^b^

^a^Mann–Whitney test

^b^Fisher’s exact test

## Discussion

This retrospective analysis is one of the largest reporting patients treated for isolated popliteal artery injury at a level I trauma center. Within this cohort, the majority of patients was affected by blunt trauma with an overall amputation rate of 16% (8 out of 50 patients); however, the MESS did not predict amputation.

Whereas penetrating trauma mechanism is the leading cause of vascular trauma in military surroundings, blunt trauma represents the majority of cases in civilian series. Often accompanied with soft tissue and skeletal injuries, higher amputation rates are reported for blunt vascular trauma compared to patients suffering from penetrating trauma [5, 6, 18–21]. Despite being uncommon, traumatic lesions of the popliteal artery are associated with a considerably high risk of limb loss substantial impact on affected patients. Limb salvage should be attempted by any means but accompanying complications such as infection and sepsis with the consequent need for secondary amputation are associated with prolonged hospitalization, increase in overall costs as well as in potential life-threatening conditions [10, 12].

Our series reflects one of the largest single-center series of isolated popliteal artery trauma reporting 50 patients with 94% sustaining blunt trauma. Registry data from national trauma data bases include larger numbers of patients but reproducibility may be limited due to limited data collection compared to single-center studies. With the overlap of reported data concerning the time frame of data analysis within the same database, comparability is also limited [5, 22, 23]. With patients mainly affected by motor vehicle, sport-related or work-related accidents, our current data may better reflect trauma distribution in a European setting, compared to analyses of US data bases with more gun-related trauma.

Patients who had to undergo secondary amputation were all affected by blunt trauma, leading to an amputation rate of 17% within this subgroup. In literature regarding amputation rates in patients with isolated traumatic lesion of the popliteal artery, reported rates vary between 14.5% and 36.0% [5, 6, 21, 24–26] with increased frequencies in blunt trauma with up to 46.0% [5, 6, 21]. Therefore, the overall in-hospital amputation rate of 16% within our series represents a relatively low rate compared to reported data in literature.

The currently available scoring systems have the intention to predict the need for primary amputation in extremity trauma. All of them have in common that the presence of shock and ischemia as well as varying information about injuries of the surrounding tissues are included, leading to a defined threshold predicting limb salvage and amputation [8–11]. The only score based not solely on retrospective but also on prospective data is the MESS which is the most commonly used scoring system [12]. The largest cohort of patients prospectively evaluated with MESS, PSI, LSI, and NISSA was published by Bosse et al. [27]. The authors concluded that none of the evaluated scores should be used for the decision if an extremity should be primarily amputated or not as the sensitivity for the prediction of amputation is low in all scores. Regarding the specificity of low scores to predict limb salvage, the evaluated scoring systems were described to be useful; however, none of them was able to predict functional outcome [15, 27]. Regarding retrospective analysis of mangled extremities, MESS was reported to be able to predict amputation but the authors concluded that the decision on primary amputation should not be based on the scoring alone as several patients with a MESS > 7 had good functional outcome after successful revascularization of affected limbs [16]. Therefore, authors concluded that limb salvage should be attempted by any means [15, 16]. The MESS was introduced to predict primary amputation and failed to do so. Regarding our data, in a cohort of patients undergoing revascularization following popliteal artery trauma, the initial MESS did not predict outcome regarding limb salvage. Demonstrating a significantly prolonged length of hospital stay in the group of patients undergoing amputation with potential systemic complications following a “limb salvage by any means” strategy, a tool to predict these clinical courses is needed. Within the amputation group (details see Table 4), the indication for amputations was mainly infectious complications, both local and systemic, but MESS was not able to predict these clinical courses as amputation rates were similar in this civilian cohort of mainly blunt trauma (AMP MESS 8.4 ± 4.1 vs. LS 8.1 ± 3.8; *p = *0.830). Interestingly, there was a significantly higher overall rate of fasciotomies performed within the group of patients suffering severe trauma (MESS ≥ 7.93% vs. 59%, *p = *0.006). Therefore, it seems that fasciotomy potentially prevented the need for lower limb amputation in patients suffering severe lower limb trauma in our patient cohort. Fasciotomy is performed to prevent compartment syndrome following trauma and reperfusion after ischemia. Aiming to prevent irreversible muscle damage, medial and/or lateral incision of the fascia of the four anatomical compartments of the calf muscles is performed. The most important risk factor for the development of compartment syndrome following ischemia is the time until revascularization is achieved [28]. Despite this, primary fasciotomy is still controversial, as the procedure itself is associated with the need for re-interventions, until definite wound closure is achieved. The increased rate of fasciotomy in severe trauma also correlates with the reported predictors for amputation within our series (Table 2). The maximum level of myoglobin as a sign of muscle damage as well as a decrease in kidney function, defined as minimum of eGFR and, respectively, the need for hemofiltration as signs of acute kidney injury, following revascularization demonstrate the importance of an adequate management of reperfused muscle tissue following ischemia to prevent secondary amputation. Two other factors, namely concomitant venous trauma and classification of ischemia severity showed a statistical trend to correlate with amputation, yet did not reach statistical significance at *p* values of 0.0591 and 0.0638, respectively. Speculatively, analysis of a larger cohort may demonstrate a significant influence of further predictors.

**Table 4:** Details on clinical course of patients undergoing secondary amputation

Nr	Age, sex	Type of trauma	MESS	Shock	Concomitant injuries	Vascular reconstruction, fasciotomy	Clinical course	Level of amputation (days since revascularization procedure)	Systemic complications
1	78, m	Work related (fall from 6 m height)	5	No	Tibial fracture with knee luxation	Venous interposition graft, primary fasciotomy	Muscle necrosis, superinfection (Enterococcus faecalis)	Exarticulation knee joint (15)	Acute kidney injury with temporary hemofiltration
2	47, m	Motor vehicle (motorbike)	10	No	Dislocated fracture of the proximal tibia and fibula	Venous interposition graft, primary fasciotomy	Muscle necrosis, superinfection	BTK (21)	Sepsis
3	31, m	Motor vehicle (motorbike)	8	No	Fracture of the distal femur, fracture vertebral body Th11, pulmonal contusion, laceration liver	Venous interposition graft, primary fasciotomy	Vascular re-intervention due to thrombosis of the graft, muscle necrosis, superinfection	Exarticulation knee joint (24)	Acute kidney injury without hemofiltration, sepsis
4	35, m	Work related (coverage of both legs with hard dirt)	6	Yes	Fracture lateral condyle of the femur and fibula	Venous interposition graft, primary fasciotomy	Muscle necrosis, superinfection	BTK (24)	Acute kidney injury with temporary hemofiltration, sepsis
5	32, m	Motor vehicle (motorbike)	6	No	Tibial fracture with knee luxation	Venous interposition graft, primary fasciotomy	Vascular re-intervention due to thrombosis of the graft, muscle necrosis, superinfection	ATK (18)	Acute kidney injury without hemofiltration
6	33, m	Motor vehicle (motorbike)	14	No	Tibial fracture with knee luxation	Venous interposition graft, primary fasciotomy	Muscle necrosis, superinfection	ATK (17)	Acute kidney injury with temporary hemofiltration, multi organ failure, sepsis
7	31, m	Work related	12	No	Soft tissue trauma	Anastomosis of disrupted popliteal artery and vein, secondary fasciotomy	Muscle necrosis, superinfection	ATK (103)	None
8	31, m	Motor vehicle (motorbike)	6	No	Tibial fracture with knee luxation	Venous interposition graft, primary fasciotomy	Muscle necrosis, superinfection	ATK (74)	None

*ATK* above the knee; *BTK* below the knee; *m* male; *MESS* Mangled Extremity Severity Score

This study is limited by its retrospective character as a retrospective analysis is always as good as the documentation system is. Even though the presented cohort of 50 patients with an isolated popliteal artery injury can be considered substantial given the rare nature of the clinical pattern, analysis of a larger cohort may identify further predictors of limb salvage or loss. As reported in literature, a shortening of time of ischemia is one of the key factors to reduce risk of amputation. Temporary intravascular shunting (TIVS) has gained importance especially in military scenes, and it is still discussed controversial in civilian trauma [29]. In our center, TIVS is not established and not used; therefore, within our series, none of the patients was treated with TIVS, irrespective of trauma severity.

In conclusion, in our cohort of patients with no primary amputations, a MESS > 7 did not predict delayed amputation. Regarding predictors for amputation, beside elevated myoglobin levels and renal insufficiency, interestingly, combined arterial and venous injury of the popliteal area showed a trend towards higher amputation rates. Importantly, revascularization and soft tissue protection by generous fasciotomy should always be attempted, as this approach is likely to succeed and increase limb salvage rates.

### Supplementary Information

Below is the link to the electronic supplementary material.

Supplementary file1 (DOCX 14 kb)

## Data Availability

The authors confirm that the data supporting the findings of this study are available within the article and its supplementary materials.
