# Supplementary material for: The Mangled Extremity Severity Score (MESS) does not predict amputation in popliteal artery injury
Source: Eur J Trauma Emerg Surg. 2022 Nov 30;49(6):2363–71. doi: 10.1007/s00068-022-02179-4 (PMC10728240; doi:10.1007/s00068-022-02179-4)
Supplement: Supplementary file 1 — Supplementary file1 (DOCX 14 kb) [file 68_2022_2179_MOESM1_ESM.docx]

Table xxx: Examined predictors for the occurrence of amputation

| Predictor | Subcategories | | Levels |
| --- | --- | --- | --- |
| Sex |  | | male, female |
| Age |  | | [years] |
| Mechanism of Trauma |  | | traffic, work, sports |
| Type of trauma |  | | blunt, penetrating |
| Vascular trauma type |  | | dissection, disruption, occlusion, partial lesion |
| Popliteal segment |  | | Above the knee, below the knee |
| Vascular revision |  | | yes, no |
| Ischemia time >6h |  | | yes, no |
| Delayed revascularization (>12 h after trauma) |  | | yes, no |
| Concomitant injury | **Bone injury** | | yes, no |
|  |  | **Luxation** | yes, no |
|  |  | **Fracture** | yes, no |
|  | **Venous injury** | | yes, no |
|  | **Neural injury** | | yes, no |
| Fasciotomy |  | | yes, no |
| Rutherford category of ischemia |  | | I, IIa, IIb, III |
| MESS Score |  | | continuous |
| Myoglobin [maximum posttraumatic] |  | | [nMol/L] |
| Creatinine kinase [maximum posttraumatic] |  | | [U/L] |
| Acute kidney injury |  | | yes, no |
| eGFR [minimum posttraumatic] |  | | mL/min/1.72m^2^ |
